# Improving Interoperability in ePrescribing

**DOI:** 10.2196/ijmr.2089

**Published:** 2012-11-22

**Authors:** Sten-Erik Öhlund, Bengt Åstrand, Göran Petersson

**Affiliations:** 1 eHealth InstituteLinnaeus UniversityKalmarSweden; 2 Information SystemsDepartment of Management and EngineeringLinköping UniversityLinköpingSweden; 3 Logica Sverige ABKalmarSweden

**Keywords:** eHealth, Electronic prescribing, Electronic prescription, Information quality, Interoperability

## Abstract

**Background:**

The increased application of eServices in health care, in general, and ePrescribing (electronic prescribing) in particular, have brought quality and interoperability to the forefront. The application of standards has been put forward as one important factor in improving interoperability. However, less focus has been placed on other factors, such as stakeholders’ involvement and the measurement of interoperability. An information system (IS) can be regarded to comprise an instrument for technology-mediated work communication. In this study, interoperability refers to the interoperation in the ePrescribing process, involving people, systems, procedures and organizations. We have focused on the quality of the ePrescription message as one component of the interoperation in the ePrescribing process.

**Objective:**

The objective was to analyze how combined efforts in improving interoperability with the introduction of the new national ePrescription format (NEF) have impacted interoperability in the ePrescribing process in Sweden, with the focus on the quality of the ePrescription message.

**Methods:**

Consecutive sampling of electronic prescriptions in Sweden before and after the introduction of NEF was undertaken in April 2008 (pre-NEF) and April 2009 (post-NEF). Interoperability problems were identified and classified based on message format specifications and prescription rules.

**Results:**

The introduction of NEF improved the interoperability of ePrescriptions substantially. In the pre-NEF sample, a total of 98.6% of the prescriptions had errors. In the post-NEF sample, only 0.9% of the prescriptions had errors. The mean number of errors was fewer for the erroneous prescriptions: 4.8 in pre-NEF compared to 1.0 in post-NEF.

**Conclusions:**

We conclude that a systematic comprehensive work on interoperability, covering technical, semantical, professional, judicial and process aspects, involving the stakeholders, resulted in an improved interoperability of ePrescriptions.

## Introduction

The increased use of eServices in health care in general, and ePrescribing (electronic prescribing) in particular, has placed a focus on quality [1] and interoperability [2]. In Sweden, ePrescribing has increased notably during the 2000s. In 2011, ePrescriptions constituted more than 90% of all filled prescriptions [3].

### ePrescribing

**Figure 1:**
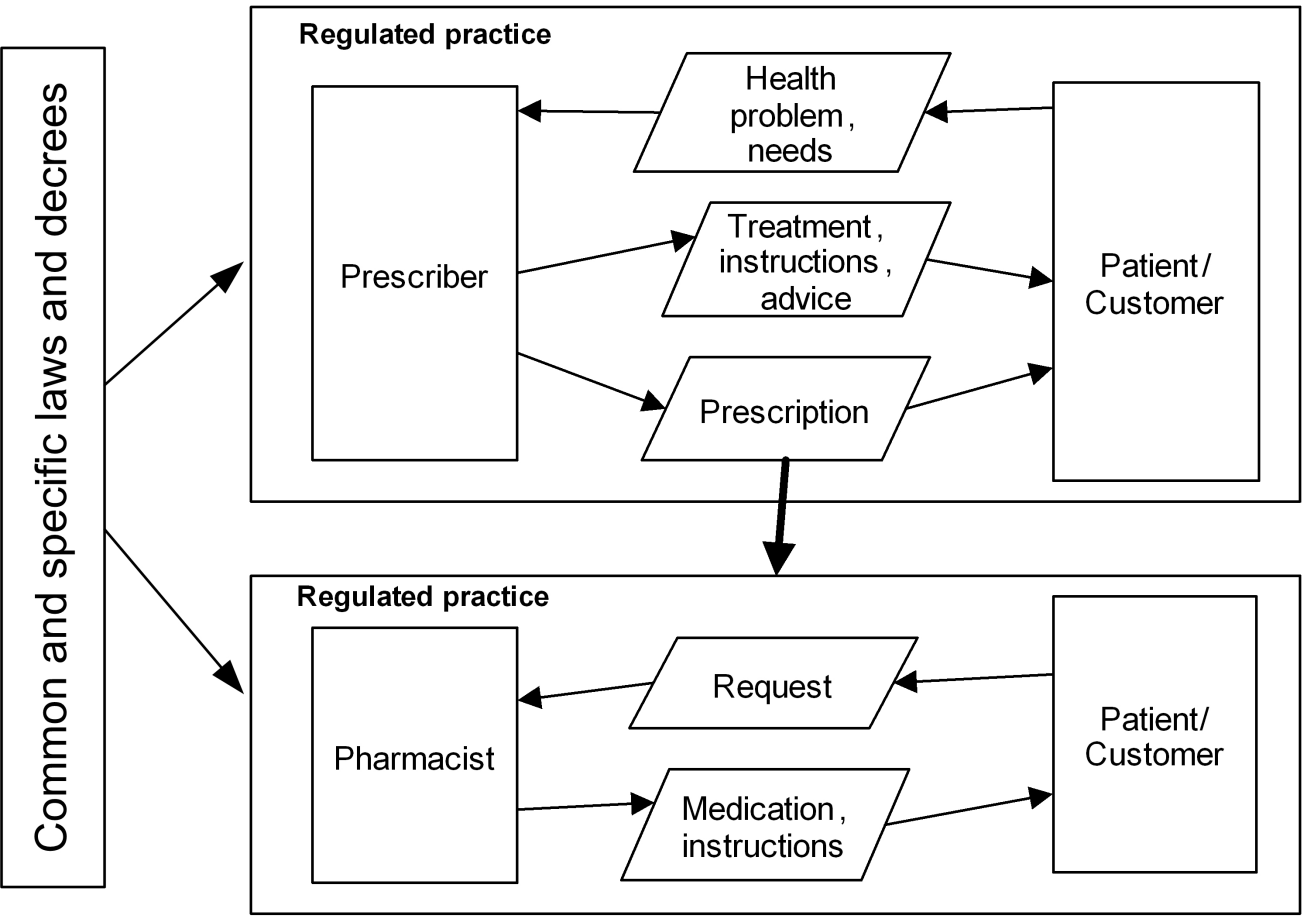
The Generic Regulation Model applied to ePrescribing [4].

ePrescribing is a co-operation between prescriber, patient, and pharmacist for the purpose of medical treatment of the patient. A prescription is a regulated, social act, authorizing a pharmacist to dispense a medical drug to a patient [4]. ePrescribing has been analyzed using a Generic Regulation Model (GRM) (Figure 1) [4,5].

The interaction between the patient and the prescriber results in a prescription having multiple functions, such as an authorization to a pharmacy to dispense a medical drug according to certain rules, a directive to the patient for medical treatment, and a commitment on behalf of the health care organization (a reimbursement commitment specific to Sweden) to the patient that the patient will receive reimbursement for the prescribed medical drug. The prescription also provides a basis for further information processing by authorities and researchers.

### Stakeholders in ePrescribing

**Figure 2:**
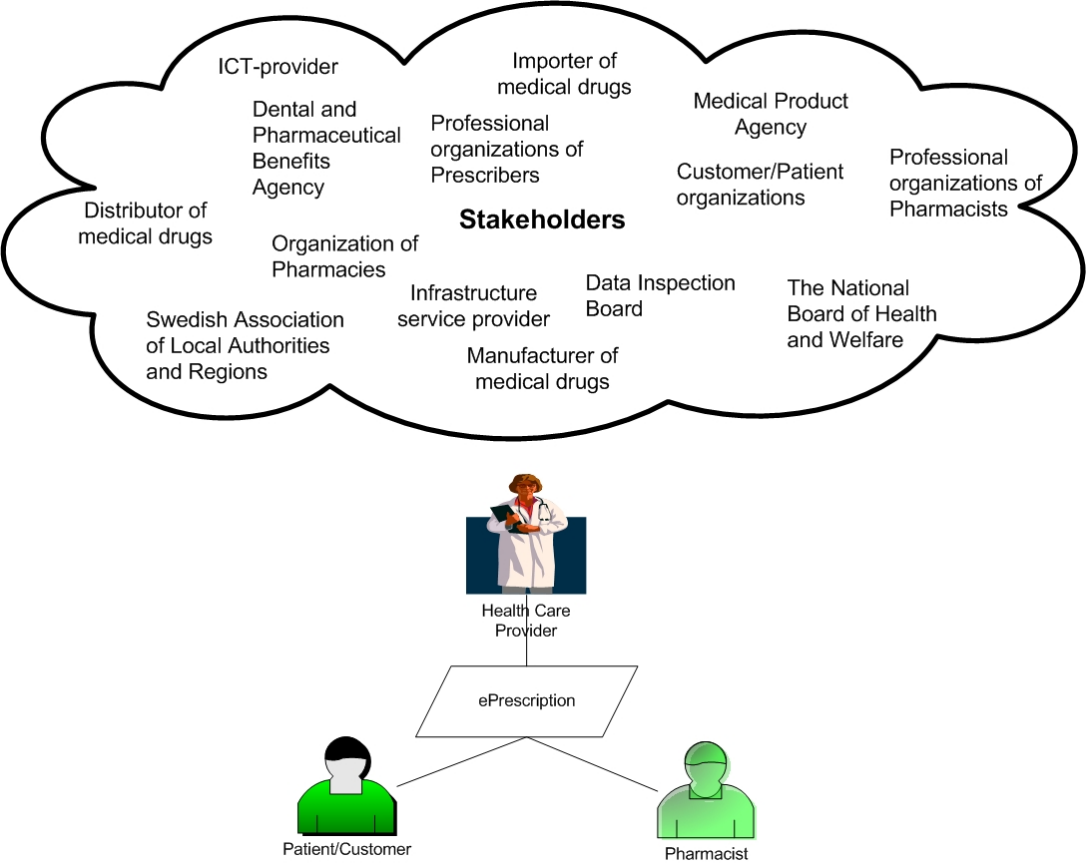
Stakeholders in ePrescribing.

The ePrescribing process involves a large number of stakeholders having interest in, being influenced by, and who influence the ePrescribing process and the content of the ePrescription (Figure 2). The multiple functions inherent in the prescription and the different stakeholders involved make the communication regarding ePrescriptions complex. As an illustration, the Medical Products Agency approves the medical drugs to be sold on the Swedish market, determines which drugs are to be considered generic exchangeable drugs, and acts as the statutory authority for prescription regulations; whereas, the Dental and Pharmaceutical Benefits Agency decides which medical drugs are to be reimbursed and determines procurement and sales prices. Furthermore, the National Board of Health and Welfare regulates the health care sector and provides information about prescribers and their prescription rights, as well as granting practitioner licenses to prescribers and pharmacists. Basic information about the medical drugs to be prescribed is provided by the Medical Product Agency and by the manufacturers and importers of medical drugs. This information is communicated both to the prescriber organizations and their Electronic Health Record (EHR) systems, and to the pharmacies and their dispensing systems via a state-owned infrastructure service provider. This service provider also provides e-services for ePrescriptions, dispensing, reimbursement of prescriptions, and statistics.

### ePrescribing as Communication

**Figure 3:**
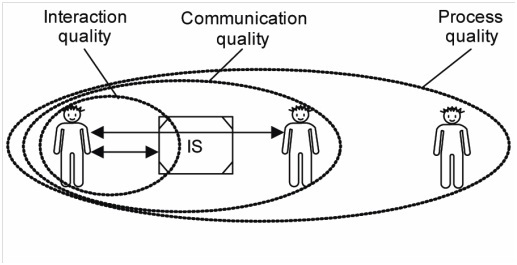
Different layers of quality according to the Information Systems Actability Theory [6].

Information Systems Actability Theory [6] considers an Information System (IS) to be an instrument for technology-mediated work communication. In addition to the technical aspects, user interaction, communication between users applying IS as an instrument of communication, and the overall influence of information and actions involved in the process, impact the ePrescribing process. The ability to interoperate in ePrescribing is closely related to interaction, communication, and process quality (Figure 3).

A sender interacts with an IS to communicate something to a receiver, which in turn interacts with an IS to read and interpret a message. Interaction quality criteria can be defined for this activity, ie, what the users are doing *with* the system. In this process, there is also communication between the sender and the receiver about what the users are doing *through* the system. Communication quality criteria referr to the formulation and communication of messages by a sender, as well as the reading and interpreting of messages by a receiver. Process quality is concerned with what the users do *outside* the system, ie, the effects of IS usage on work practice.

Interaction quality criteria for a prescriber might imply that the vocabulary of the system is intelligible and in line with terminology of the profession or regulated practice—that it is obvious what the user can do in the system and that consequences of different actions are transparent. For a prescriber, this could imply that the consequences approving or cancelling a prescription are clear and that navigation between the various parts of the system is easy.

For a pharmacist, communication quality criteria might refer to relevant prescription information being easily available for dispensing, that the information is accurate, that it is obvious who the sender is, and that the intention of the prescriber is unambiguous.

In general, process quality criteria refer to the requirement that the information from the system is useful on behalf of its users; ie, that the information has a meaningful use. In the process of ePrescribing, eg, the system should support process objectives, such as patient safety, correct reimbursement processes, clear instructions for the patients, but should also support the objectives of other stakeholders, such as achieving correct statistics for researchers and authorities.

**Figure 4:**
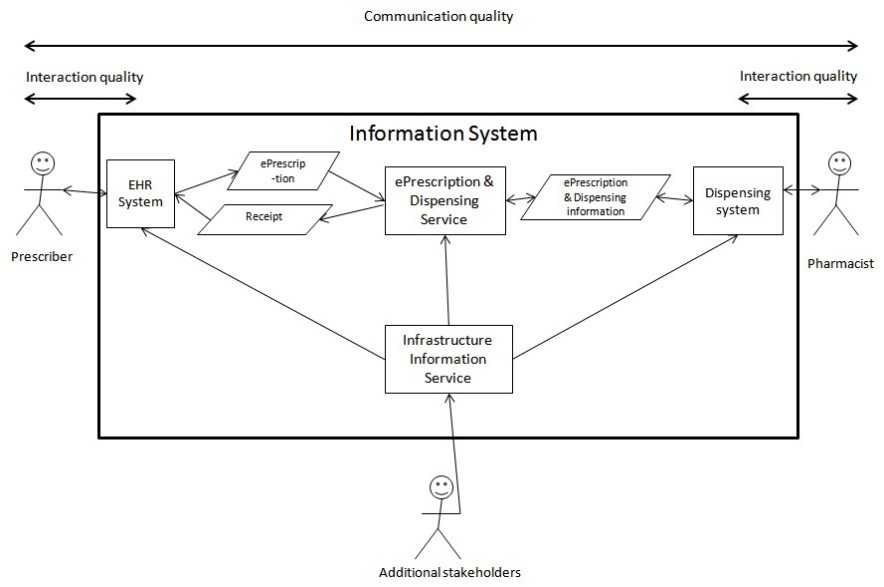
Overview of IS and stakeholders involved in ePrescribing communication.

The quality of the communication between prescriber and pharmacist is dependent on many factors, among them the quality of the communicated message. The quality of the communicated message is dependent on the quality of formulating an ePrescription (part of communication quality) and by interaction quality of the EHR system. Finally, how the communicated message is presented and made available to the pharmacists in their dispensing systems affects the overall communication quality. Here, we elucidate the communication of ePrescription messages between the EHR systems prescription modules, the ePrescription service system, and the quality of this message (box marked IS) with regard to the requirements that have been established for this communication (Figure 4). In this study interaction, quality and communication quality aspects in EHR and dispensing systems are not considered, although they affect the quality of the communicated message.

### Communication Quality in ePrescribing

The quality of the ePrescription message is one part of communication quality in the ePrescribing process between the prescriber and the pharmacist. The stakeholder’s involvement in providing infrastructure information influences the communication quality, such as the information about the population, medical drugs, prices, reimbursement rules, prescribers, and pharmacist rights. This information is provided through various information services, which can be grouped into infrastructure information services, and ePrescription and dispensing services. These services are provided to the prescriber and pharmacy systems and have an important influence on the communication quality. The interaction quality of the EHR and dispensing systems are important to consider in assessing communication quality [7].

### Interoperability in ePrescribing

The various definitions of interoperability involve different perspectives on interoperation. Some definitions focus on the ability of systems to interoperate [8], and others focus on the ability of people to interoperate (individual, organizational level) by using systems to achieve a certain goal [9,10].

We regard interoperability as the capability of the entire process, involving people, systems, procedures, and organizations, to interoperate using IS in order to achieve its objectives. Here, we focus on one vital aspect of the interoperation in the ePrescribing process: the communication of the ePrescription message. To understand the complexity of the ePrescription message, it is necessary to analyze it as one component of communication in the ePrescribing process involving many stakeholders and IS. Thus, the content of the ePrescription message is dependent on stakeholder involvement both in the infrastructure services and in the actual formulation and interpretation of the prescription message by prescriber, pharmacist, and systems involved.

European Interoperability Framework (EIF) [10] defines four levels of interoperability: legal, organizational, semantic, and technical interoperability (Table 1).

**Table 1:** Levels of interoperability.

Level of interoperability	Description
Legal	Alignment of legislation concerning the interoperation between different organizations, which affects how and what can be communicated
Organizational	How different organizational processes are integrated and how information exchange is managed
Semantic	Processing of information in a meaningful way, provided that information in the communicated message is precisely defined, agreed, and understood by all the stakeholders involved
Technical	Technical prerequisites linking different systems, such as communication protocols, message format, services, interface specification, etc.

All these levels of interoperability influence the actual implementation of the ePrescription message and service, which is the central point where the interoperation is achieved. To achieve interoperability, significant emphasis in eHealth has been put on the application of standards and common terminology, such as ISO 13606 [11], HL7 [12], and Snomed CT [13]. Although these standards are important building blocks in achieving interoperability, less attention has been put on other factors for improving interoperability, such as measuring interoperability and stakeholder involvement in order to address other aspects of interoperability, which is particularly relevant for interorganizational interoperability.

### The Implementation of a New National ePrescription Format in Sweden

The introduction in 2009 of a new National ePrescription Format (NEF) in Sweden was the result of an effort to improve interoperability in ePrescribing. Consequently, this provided a unique opportunity to study NEF’s effect in terms of changes on the interoperability in ePrescribing.

The implementation of ePrescription messages sent from health care organizations to pharmacies in Sweden has evolved during three decades. Varying communication standards with different message specifications (based on the UN standard MEDPRE [15] and the pre-standard ENV 13607 [7]) have been introduced and applied. The infrastructure has constantly evolved, from point-to point message communication of prescriptions by local health care organizations to local pharmacies, towards centralized communication on both sides. ePrescription have evolved from a mere electronic transfer of a message to management of the prescription during its entire life cycle with repeated refills. A growing number of health care regions have been involved and the number of prescription systems has increased. In 2009, there were 16 EHR-systems with ePrescription modules and one web-based prescription system sending ePrescriptions from 21 health care regions in Sweden.

Interoperability problems in ePrescribing were observed in an increasing number of issues by the support team at the Apoteket AB (National Corporation of Swedish Pharmacies—the state-owned pharmacy chain, which, at that time, was a monopoly). With the increased volume of ePrescriptions forecasted in 2006, from 30% to 80% (of new prescriptions) during the subsequent years, the handling cost of poor quality was expected to increase considerably. There was no automated control of incoming prescriptions other than a failure to store the ePrescription in the database. The system did not report any information about the possible cause of errors. Testing and approval of EHR systems to send ePrescriptions were rudimentary, and the process and organizational aspects of this were unclear, as was the process for maintenance and development of the ePrescribing process. Focus was on managing the technical communication, while the process of how to handle errors in content was pushed to the pharmacist at the end of the process.

To meet these challenges, a joint project between Apoteket AB and the regional health care providers was initiated in 2006 with the purpose to improve patient safety and to decrease the cost of deficient quality in ePrescribing. The project subsequently implemented the new NEF-format [14] in Sweden, together with a stricter test procedure than previously. Also, the automatic control of format and prescription rules was introduced. From May 31, 2009, all ePrescriptions in Sweden were issued in the NEF-format.

### Objective

The objective of the present study was to analyze the manner in which the combined efforts in improving interoperability with the introduction of NEF affected interoperability in the ePrescribing process in Sweden, with a focus on the quality of the ePrescription message.

## Methods

The present study was an intervention study. The intervention consisted of the combined efforts in implementation of NEF. We measured interoperability problems prior to and after the intervention: pre-NEF and post-NEF.

In the pre-NEF study period, interoperability errors in pre-NEF prescriptions were validated against the format specification and prescriptions rules valid for the pre-NEF study period. In the post-NEF study period, interoperability errors in post-NEF prescriptions were measured against the format specification and prescription rules valid for the post-NEF study period. The prescription rules did not change between the study periods.

In the two study periods, we compared changes in adherence to the agreed format specification and prescription rules based on legislation and agreed praxis. Consequently, the focus was on communication quality between health care and pharmacy using ePrescription as an instrument of communication for the medical treatment of a patient. Also, the assessment of communication quality was limited to the formal and documented requirements on the ePrescription message.

The hypothesis was that adherence to the agreed format specification and prescription rules should be improved with the introduction of NEF, resulting in fewer interoperability errors in the post-NEF period.

Information System Actability theory and theories about interoperability were used to analyze the ePrescribing process, the implementation of NEF, and its results.

### Design

Consecutive sampling was applied on all incoming ePrescriptions during two periods: April 3, 2008, to May 3, 2008 (pre-NEF), and April 3, 2009, to May 3, 2009 (post-NEF). To be able to demonstrate a significant change of 1% between the two study periods, the required sample size was estimated to be approximately 1,450,000. The calculation was made by using a sample size calculator developed by Rollin F. Brant [15].

Consecutive sampling of all ePrescriptions during the two study periods was considered to be the best choice for handling historic changes in the drug database, including all ePrescriptions during the study periods. Electronic Data Interchange For Administration, Commerce and Transport (EDIFACT) prescriptions were present only in the pre-NEF period and were, therefore, excluded from the study.

During the two study periods, all of the prescribing systems used in Sweden were expected to be represented with a fair amount of ePrescriptions. During one month of sampling, ePrescriptions from the majority of the prescribers would be represented, except those making only a few prescriptions per year.

### Definition of the ePrescribing Process

**Figure 5:**
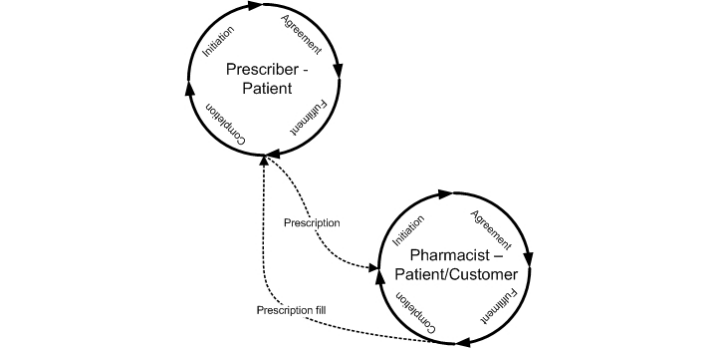
ePrescribing process seen as a process of initiation, agreement, fulfillment, and completion in two exchange situations: Prescriber – Patient and Pharmacy – Patient/Customer.

In this study, ePrescribing was regarded as a process that starts with a communication between prescriber and patient and that is related to medical treatment. The process also includes communication between patient and pharmacy, followed by the completion phase with the aim to evaluate the result of the medical treatment. The ePrescribing process is described and further developed in [4] as two interconnecting loops of generic phases following the Generic Exchange Model (GEM) [5]: initiation, agreement, fulfillment, and completion (Figure 5). Thus, ePrescribing is a complete process for medical treatment using ePrescriptions as an instrument for communication to achieve the process objectives.

In this study, we analyzed the interoperability problems in the ePrescription communication in ePrescribing. We did not study other interoperability problems in ePrescribing connected with or related to actions and consequences in the stakeholder processes influencing the medical treatment of the patient.

A prescription set contains a number of prescriptions for a patient by a prescriber at a certain point in time. A prescription set is equal to a prescription message. A prescription refers to the prescription of a medical drug.

### Intervention: Combined Efforts for Implementation of NEF

The intervention refers to all of the combined efforts associated with the implementation of NEF (Table 2).

**Table 2:** Summary of actions taken with the introduction of NEF.

Action	Description
Phasing out of old formats	Phasing out of the United Nations Electronic Data Interchange For Administration, Commerce and Transport (UN/EDIFACT) format.
Definition of terms	Extensive definition of the usage of terms in the ePrescription message with minimal changes in the previous ePrescription message.
New features in ePrescription	New features of the ePrescription message: unique prescription identifications, version, and name of EHR system.
Format control	Applying a new strict Extensible Markup Language (XML) schema complying with the ePrescriptions format to validate incoming ePrescriptions.
Validation of prescription rules	“Online” validation of prescription rules and the completeness of ePrescriptions in the communication process.
Improved feedback to prescriber	New, improved feedback from the pharmacy systems to the prescriber, including validation results.
New test and approval procedures	Applying new and more rigorous test procedures before approving an EHR system for the sending of ePrescriptions to a pharmacy.

During the intervention, stakeholders from the health care providers, pharmacies, and software vendors were involved. The stakeholders participated in the work to achieve a more rigorous and developed message specification, an improved testing and approval process, the implementation of automatic control of incoming prescriptions messages, and to secure a more developed feedback mechanism, using a new extended acknowledge receipt message with status and error codes.

There were common national and regional meetings, including a referral and revision procedure of the NEF documentation. The new format specification was clarified with supplementary documentation regarding the interpretation of the specification.

Regarding the scope of the testing of the EHR-systems, it was decided to focus on the communicated message and that the tests included quality controls in the EHR-systems of prescriptions before being approved and sent. The implementation plans of the regional health care centers, Apoteket AB, and EHR vendors, were coordinated.

An important question was how to manage the different errors identified in the new process by the various stakeholders. Three different overall validation status of the ePrescription were defined: Accepted, Rejected, and Accepted with warning.

Another question that was addressed was the manner in which the health care regions should handle and communicate the rejection of a prescription. A third question pertained to the appropriate and legal actions to be taken by the pharmacist in handling ePrescriptions with errors that are accepted with warning. The messages linked to each error status code were also discussed extensively and were revised to be sufficiently comprehensive so as to be directly communicated to the prescriber.

One major challenge was to manage all the 16 EHR-systems to be changed, tested, and approved for the new format. Apoteket AB and the health care regions made a common effort to put pressure on the software vendors and on the health care regions to provide implementation plans to be able to implement NEF in time.

One difficult issue was deciding which organization should be responsible for administration and coordinating the development of the ePrescribing process. The test and approval process of the EHR systems were clarified, with the right to appeal for software vendors to the project organization if they were not satisfied with a decision. Both these tasks were at the end taken by a state-owned infrastructure company.

Overall, the intervention can be regarded as an effort to improve interoperability on four levels: legal, organizational, semantic, and technical (Table 1).

### Analysis of Interoperability Problems in ePrescription Communication

The analysis of the ePrescription messages was made applying a specifically developed software that analyzed all collected ePrescriptions sent to Apoteket AB in Sweden during the two study periods. In brief, a test procedure was set up in the two study periods accessing all sampled ePrescriptions in the former XML-format (pre-NEF) and in the new NEF XML-format (post-NEF) respectively [2]. The collected electronic prescriptions were validated on the basis of an XML-schema that agreed with the previous format specification (pre-NEF) and according to the new NEF (for the post-NEF prescriptions) and then against the prescriptions rules implemented in the software.

The prescription rules implemented in the software were derived either from legislation or agreed praxis between the parties of the exchange. The prescription rules were validated by pharmacists and legal experts. The legal prescription rules did not change with the introduction of NEF. With NEF, prescription rules were formalized in a way suitable for implementation of controls of ePrescriptions. A control of the availability of the drugs on the Swedish market at the time of prescribing was undertaken using a historical drug database built for the selected test periods of approved and marketed drugs in Sweden.

No information about patients or prescribers, or any information that could be traced back to an individual was collected from the prescriptions. No controls were made of the medical content or adequacy of the prescription.

### Classification of Errors

Two major classes of errors were identified: format errors and prescription rule errors. Format errors consist basically of valid terms and structure, data type errors, enumeration code errors, and structural errors (sequence, mandatory information, and cardinality). Format errors correspond to semantic interoperability errors (valid terms, codes, structures), where syntactic interoperability errors are the major part. The format errors were more precisely defined as a deviation from the XML-schema reflecting the different format specifications. For the post-NEF sample, the published XML-schema from the NEF-project was applied. For the pre-NEF sample, an XML-schema was constructed on the same basis and principles as the NEF-schema, but this schema adhered to the pre-NEF format specification. Format errors captured in the study are summarized in Table 3.

**Table 3:** Summary of format errors captured in ePrescriptions.

Format errors	Description
Incorrect code enumeration	Incorrect qualification code according to format specification
Element not defined in the specification	XML-tags not defined in the specification
Incorrect sign or format	Violating pattern constraints, such as using forbidden characters or wrong date-format
Override of maximum length	Excessive number of characters in a given field
Incomplete structure	Missing mandatory fields in a structure
Invalid data type or missing values	Incorrect data type or missing values in field (minimum length, minimum value, missing value)

Prescription rule errors were defined as legislative rules for a correct and complete prescription and also rules for agreed praxis for handling reimbursement rules, rules for communicating to the pharmacy in special cases of identification of the patient, correct references to drug identity, and valid packages for prescribed drug. Prescription rule errors correspond to legal, organizational (process), and semantic interoperability errors.

In all, 24 prescription rules were implemented and used in the validation. Prescription rule errors captured in the study are summarized in Table 4. Certain prescription rules are aggregated into one rule or collection of rules for improved readability. See Appendix 1 for a description of prescription rules used and actions taken when errors occur.

**Table 4:** Summary of prescription rule errors captured in ePrescriptions.

Prescription rule errors	Description
Incomplete prescriber information	Missing name, address, or telephone number
Invalid prescriber code	Incorrect format on the prescriber code
Missing workplace code	Without workplace code. The prescription can be dispensed only if the customer pays the full price for the medical drug.
Invalid reimbursement status for prescribed drug	The prescriber (or the system by default) has affirmed that the prescribed drug is valid for reimbursement, when the drug in question is not a reimbursement drug.
Incomplete or erroneous patient information	For example that the personal identification number is incorrect, or the name is missing.
Invalid drug identity	The drug identity in the prescription is not found in the database of approved and marketed drugs in Sweden at the point of issue of the prescription.
Prescription not valid for controlled substances	The prescription does not follow the specific prescription rules for these types of drugs.
Invalid combination of packages	The packages combined in the prescription for a multiple choice of a prescribed medical drug is not of the same medical product according to the drug database.
Missing directions for patient use	Text is missing when a medical drug is present in the prescription.

### Statistics

Descriptive statistics were generated from databases using SQL-queries. Pearson chi-square, uncorrected for continuity, was calculated to test that no change in interoperability errors occurred. A high number of Pearson chi-square would indicate a significant improvement in interoperability in the post-NEF sample. *P*<.05 was regarded to be significant.

## Results

### Sampled Prescriptions

The pre-NEF sample comprised 1,270,399 prescription sets. The number of prescriptions (prescribed drugs) was 1,910,982. The mean number of prescribed drugs in each prescription set was 1.50. The post-NEF sample comprised 1,479,588 prescription sets. The number of prescriptions (prescribed drugs) was 2,204,444. The mean number of prescribed drugs in each prescription set was 1.49 (Table 5).

**Table 5:** Sampled prescriptions—pre-NEF and post-NEF.

Prescriptions	Pre-NEF^a^	Post-NEF
Prescription sets	1,270,339	1,479,588
Prescriptions	1,910,982	2,204,444
Mean prescribed number of prescriptions per prescription set	1.5	1.5

^a^ EDIFACT prescriptions were excluded.

### Dispensing Fills and Refills

According to Swedish prescription rules, the prescriber may assign, for each prescribed drug, the number of dispensing fills and refills allowed during one year. The most common case was one single fill; the second most common included four fills/refills indicating that this represents the usual treatment for one year.

### Prescribed Reimbursement

The majority (95% pre-NEF and 92% post-NEF) of the prescriptions were asserted by the prescriber to be valid for reimbursement (Table 6).

**Table 6:** Number of prescriptions with prescribed reimbursement and mean prescribed reimbursement per prescription in pre-NEF and post-NEF samples.

	Pre-NEF		Post-NEF	
Reimbursement type	No.	Mean	No.	Mean
With reimbursement	1,810,942	94.8	2,022,957	92.8
Without reimbursement	94,971	5.0	181,487	8.2
Incorrect or missing value^a^	4225	0.2	0	-

^a^ Prescriptions in the pre-NEF sample that either used old classification codes for a reimbursement type that was no longer valid or that had a missing value.

### Errors per Prescription and Prescription Set

The following is a summary of the errors per prescription and prescription set (Tables 7 and 8):

- The total number of errors found in pre-NEF prescriptions was 5,970,737. The number of pre-NEF prescription sets that had at least one error was 1,253,134. The percentage of pre-NEF prescription sets with at least one error was 98.6% (1,253,134/1,270,399).
- The mean of pre-NEF prescription errors was 3.1 (5,970,737/1,910,982).
- The mean of pre-NEF prescription set errors was 4.7 (5,970,737/1,270,399).
- The mean number of errors for pre-NEF prescription sets *with* errors was 4.8 (5,970,737/1,253,134). No errors were found in 17,205 (1,270,339−1,253,134) pre-NEF prescription sets.
- The total number of errors found in post-NEF prescriptions was 13,735. The number of post-NEF prescription sets that had at least one error was 13,735.
- The percentage of post-NEF prescription sets with at least one error was 0.9% (13,735/1,479,588).
- The mean number of errors for post-NEF prescription sets *with* errors was 1.0 (13,735/13,735). No errors were found in 1,465,853 (1,479,588−13,735) post-NEF prescriptions sets. No post-NEF prescription sets that had more one error.
- The mean of post-NEF prescription errors was 0.006 (13,735/2,204,444).
- The mean of post-NEF prescription set errors was 0.009 (13,735/1,479,588).

**Table 7:** Summary of pre-NEF and post-NEF prescription and prescription set errors.

	Pre-NEF	Post-NEF
Total prescription sets	1,270,399	1,479,588
Prescription sets with error	1,253,134	13,735
Prescription sets with no error	17,205	1,465,853
Prescription sets with error, %	98.6	0.9
Mean error prescription sets	4.7	0.006
Mean error prescriptions	3.1	0.009

**Table 8:** Number of errors and mean error per prescription set.

		Pre-NEF			Post-NEF	
Error type	No. of errors	%	Mean error prescription set	No. of errors	%	Mean error prescription set
Format error	5,824,675	97.6	4.6	1273	9.3	0.0009
Prescription rule error	146,062	2.4	0.1	12,462	90.7	0.0084
Total	5,970,737	100	4.7	13,735	100	0.0093

Format errors in the pre-NEF prescriptions were the most common errors (5,824,675). Prescription rule errors in the pre-NEF sample were also common in absolute terms with 146,062 prescriptions rule errors but relatively few compared to format errors. Format errors in the post-NEF prescriptions were much less frequent compared to pre-NEF prescriptions with only 1273 errors. Format errors were relatively fewer in post-NEF compared to the pre-NEF sample. Prescription rule errors had decreased considerably to 12,462 errors in the post-NEF sample, although they had not decreased in the same proportion as format errors. Prescription rule errors have in the post-NEF sample become the most common error. To test the null-hypothesis, a chi-square test was made on the two samples (Table 9). The Pearson chi-square, uncorrected for continuity, was 2,626,673.01, *P* < .0001.

### Format Errors

The distribution of format errors in the two samples were compared (Table 10). To test the null-hypothesis, a chi-square test was made on the two samples (Table 11). The Pearson chi-square, uncorrected for continuity, was 2,673,508.8. *P* < .0001

### Prescription Rule Errors

The distribution of prescriptions rule errors were compared (Table 12). The largest improvement in the post-NEF sample was a decrease of Incorrect account number for patient fee, from 125,471 to 138. The second largest prescriptions rule error in the pre-NEF sample was decreased from 10,829 to 0 in the post-NEF sample. Errors that increased in the post-NEF sample were Invalid reimbursement status for prescribed drug, Invalid drug identity, Invalid multiple choice, Missing direction for patients use, and Local pharmacy destination required. To test the null-hypothesis, a chi-square test was made on the two samples (Table 13). The Pearson chi-square, uncorrected for continuity, was 141,147.86, *P* <.0001.

**Table 9:** Chi-square test of null-hypothesis with no significant improvement in interoperability errors.

Sample	No. of prescriptions with error	No. of prescriptions with no error	Total
Pre-NEF	1,253,134	17,265	1,270,399
Post-NEF	13,735	1,465,853	1,479,588
Total	1,266,869	1,483,118	2,749,987

**Table 10:** Number of format errors (XML-Schema validation errors) in pre-NEF and post- NEF prescriptions grouped by type of error.

Format error type	Pre-NEF	Post-NEF
Incorrect code enumeration	1,704,100	26
Element not defined in the specification	1,175,861	20
Incorrect sign or format	1,131,238	522
Override of maximum length	904,278	61
Incomplete structure	311,871	524
Invalid data type (not integer)	240,432	9
Override of minimum length	204,447	108
Override of minimum value	149,962	0
No amount in patient fee	2486	3
Total	5,824,675	1273

**Table 11:** Chi-square test of null-hypothesis with no significant improvement in interoperability.

Sample	No. of prescriptions with format error	No. of prescriptions with no format error	Total
Pre-NEF	1,252,337	18,062	1,270,399
Post-NEF	1166	1,478,422	1,479,588
Total	1,253,503	1,496,484	2,749,987

**Table 12:** Number of prescription rule errors grouped by type and the pre-NEF and the post-NEF sample.

	Pre-NEF		Post-NEF	
Prescription rule error type	No.	%	No.	%
Incorrect account number for the patient fee	125,471	85.9	138	1.1
Incomplete prescriber information	10,829	7.4	0	0.0
Invalid prescriber code	6279	4.3	425	3.4
Missing workplace code	1184	0.8	132	1.1
Invalid reimbursement status for prescribed drug	1007	0.7	7,589	60.9
Incomplete or erroneous patient information	895	0.6	7	0.0
Invalid drug identity	366	0.3	3,735	30.0
Prescription not valid for controlled substances	16	0.0	5	0.0
Invalid multiple choice	14	0.0	273	2.2
Missing directions for patient use	1	0.0	2	0.0
Local pharmacy destination required	0	0.0	156	1.3
Total	146,062	100.0	12,462	100.0

**Table 13:** Chi-square test of null-hypothesis with no significant improvement in prescription rule error.

Sample	No. prescriptions with prescription rule error	No. prescriptions without prescription rule error	Total
Pre-NEF	144,104	1,126,295	1,270,399
Post-NEF	12,172	1,467,416	1,479,588
Total	156,276	2,593,711	2,749,987

### Distribution of Errors per Prescribing System

**Figure 6:**
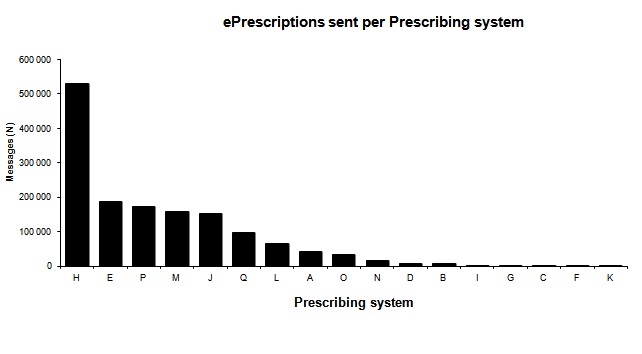
Number of ePrescription messages sent per prescribing system.

**Figure 7:**
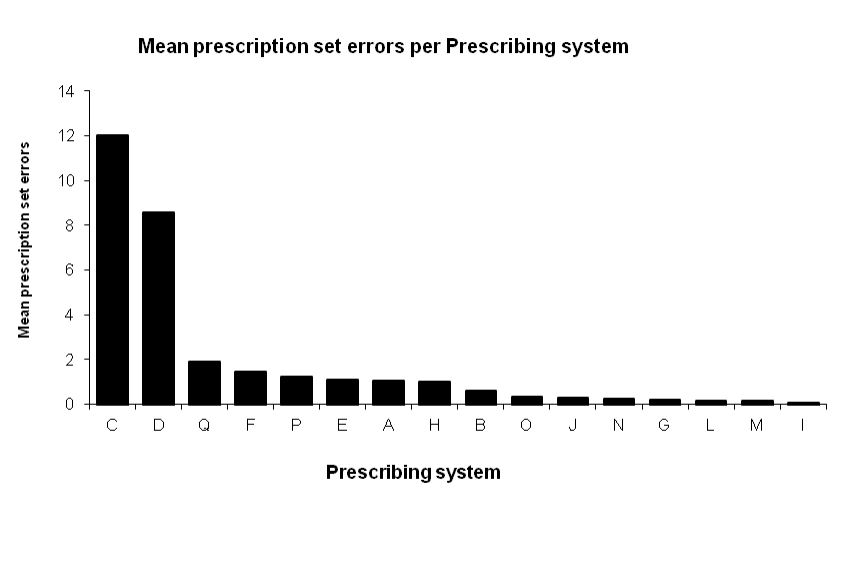
Mean prescription set errors (post-NEF) per Prescribing system.

With the introduction of NEF, the tracking of each message from the prescribing system creating the prescription was made possible*,* which was not possible with the pre-NEF sample (Figure 6). Consequently, we do not have any comparisons between the study periods. Only data from the post-NEF study are presented here. With the introduction of NEF, it was possible to measure each system’s interoperability errors (Figure 7).

### Duplicate Prescriptions

**Figure 8:**
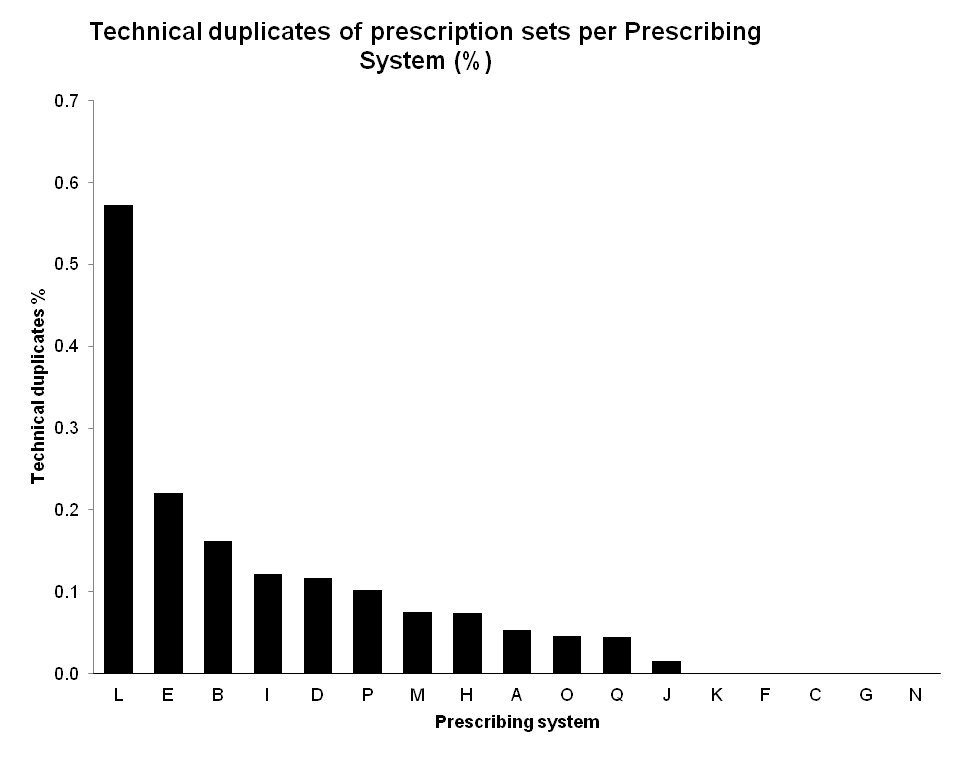
Technical duplicates of post-NEF prescription sets per prescribing system.

With the introduction of NEF, a unique identification (UUID) was introduced for each prescription, allowing rejection of the so-called technical duplicates. A technical duplicate can occur when, for example, prescriptions are being re-sent in the case of communication failures or delays. In the post-NEF, this made it possible to measure the mean number of duplicated prescriptions from different prescribing systems (Figure 8).

## Discussion

### Principal Results

The implementation of NEF substantially improved the interoperability in ePrescriptions. We have studied interoperability on four levels: legal, organizational, semantic and technical interoperability.

On the level of legal interoperability, the implementation of NEF decreased the number of ePrescriptions that were not in alignment with the legislation on prescriptions. The majority of the prescription rules errors captured concerned legal rules.

Organizational interoperability was also improved with NEF. Apart from implementing new processes such as testing and approval processes with clarification of organizational responsibilities, the process of handling certain errors was also defined, which was made possible with feedback in the acknowledge receipt. With NEF it was clarified which type of error should lead to a rejection and thus should be the responsibility of the prescriber organization to handle, and other types of errors that could be handled by the pharmacies and therefore could be accepted with a warning message. One example is the prescription rule error, missing workplace code, which was accepted with warning because it was agreed that this error, which relates to reimbursement, should not stop the dispensing of a prescription. It can be corrected by the pharmacist after contact with the prescriber*.* NEF changed the responsibilities in the process of error handling at both the prescriber and pharmacy organizations. Moreover, NEF made it possible to identify the EHR system sending a prescription allowing for a more systematic follow-up of errors to improve the ePrescribing process.

Semantic interoperability improved most, where the improvement in syntactic interoperability, which is part of semantic interoperability, was the most striking. A common and clearer definition of terms used in the message explains the improvement in the semantic interoperability. The improvement in the amount of errors like incorrect code enumeration, incomplete structure, and element not defined in the specification, have greatly benefited from a clearer format specification formalized in an improved XML-schema. Other examples of improvement in prescription rule errors are incorrect account number for the patient fee, incomplete prescriber information, invalid prescriber code, and missing workplace code. The first three errors resulted in a rejection of the prescription, the last one in an acceptance with warning. The improvement might be explained by a greater effort by EHR-vendors and by the health care regions to provide prescriptions with more complete information in order to avoid a rejection of prescriptions. In the pre-NEF situation, corrections of this kind of information were done by personnel at the pharmacies.

Another aspect of semantic interoperability is the reference to objects that are defined in the infrastructure information, such as a drug register with identities of prescribed medical drugs enabling access to attributes necessary for prescribing and reimbursement, but also workplace, pharmacy, and prescriber register. Thus, semantic interoperability depends also on information sources external to the prescription and the EHR and dispensing system. Some prescription rule errors related to infrastructure information increased in proportion in the NEF-sample such as invalid drug identity. This could be explained by more frequent changes during the sample period regarding new and withdrawn drugs in combination with low frequency of updating the drug register in the EHR system. Thus, managing infrastructure information is critical in achieving and maintaining interoperability.

The technical interoperability improved too, with phasing out EDIFACT and providing a XML-schema to improve the format controls both early in the ePrescribing process, creating the prescription and later when receiving the ePrescription to the pharmacy system. The implementation of format and prescription rule controls in the ePrescribing process, particularly at the receiving end, helped to improve the interoperability of the EHR systems. Furthermore, the control and feedback process that was implemented with NEF required a faster response from the receiving process and thus made it more beneficial to use a synchronous mode of communication, like Web services. In the pre-NEF asynchronous communication, long response time was not a problem as there was no feedback. However, with NEF, new challenges arise to provide a faster response to the prescriber, which will involve not only the ePrescription services but also the technical infrastructure in health care.

The new feature with unique identifiers made it possible to measure the number of technical duplicate prescriptions for the first time. During the sample period, the rejection of duplicates had not yet been implemented. Technical duplicates are a medical risk, which could lead to drug overdose. Improvements in technical interoperability have important effects on the overall interoperability, and vice versa, the overall requirements of semantic, organizational, and legal interoperability will influence the requirements for technical interoperability.

### Limitations

In this study, we have investigated the communication quality with regard to documented requirements on the ePrescription message before and after an intervention. The study of communication quality has addressed only that portion of communication quality concerned with the quality of the ePrescription message. Assessing the quality of EHR and pharmacy systems has not been within the scope of this study. Moreover, the effect of interoperability errors on the work practice, ie, its influence on the process quality, for example on medication errors, has not been studied. Other studies have addressed prescription errors from a pharmaceutical point of view [1,16-21].

### Future Research

There is a need to develop practical theories and methods that can assist in creating a greater awareness and understanding to address the objective of improving the interoperation between and within different sectors and organizations using IS as an instrument for communication between different stakeholders. Without theories and methods, it is easy to fall prey to technical solutions with promise of easy ways of achieving interoperability, or that good initiatives are not implemented taking into consideration the need to involve stakeholders and grasp all levels of interoperability.

In order to improve interoperability in the overall ePrescribing process, it is necessary to analyze the different information flows and the stakeholders’ roles and influence on communication and process quality. Further studies are necessary to assess the interaction quality of both prescription modules in EHR systems and prescription handling in the dispensing systems.

To improve interoperability in the ePrescribing process, the entire architecture of IS and of the stakeholders’ roles in this process need to be analyzed to assess interoperability problems and to identify areas that are important to address.

To achieve a continuous improvement of interoperability, it is necessary to establish a continuous measurement of interoperability problems as a basis for improvements. How interoperability errors influence medical errors is an important topic to study in the future.

### Conclusion

The introduction of NEF has considerably improved interoperability in electronic prescriptions in Sweden. This study showed that systematic and comprehensive work on interoperability, covering technical, semantic, professional, judicial, and process aspects, may lead to an important improvement in interoperability.

## Multimedia Appendix 1

Prescription rules and error handling.
