# Correction: Improving Interoperability in ePrescribing

**DOI:** 10.2196/ijmr.2457

**Published:** 2012-11-29

**Authors:** Sten-Erik Öhlund, Bengt Åstrand, Göran Petersson

**Affiliations:** 1 Logica Sverige ABKalmarSweden; 2 eHealth InstituteLinnaeus UniversityKalmarSweden; 3 Information SystemsDepartment of Management and EngineeringLinköping UniversityLinköpingSweden

In the recent paper by Öhlund SE et al. [Improving Interoperability in ePrescribing, Interact J Med Res 2012;1(2):e17], the percentage of prescriptions in the post-NEF (national ePrescription format) sample with errors should be 0.9% (13,735/1,479,588), rather than 0.009%. The change is minor and does not affect the overall results and conclusions of the paper. The percentage was corrected in 3 places in the paper: 1) Abstract/Results: "In the post-NEF sample, only 0.9% of the prescriptions had errors"; 2) Results/Errors per Prescription and Prescription Set: "The percentage of post-NEF prescription sets with at least one error was 0.9% (13,735/1,479,588)"; 3) Table 7 forth row: "Prescription sets with error, %       98.6      0.9". The online version on i-JMR was corrected on November 29, 2011, one week after publication, before submission to PubMed Central, Medline, or other databases.

