# Supplementary figures and images for: Improving Interoperability in ePrescribing
Source: Interact J Med Res. 2012 Nov 22;1(2):e17. doi: 10.2196/ijmr.2089 (PMC3626130; doi:10.2196/ijmr.2089)

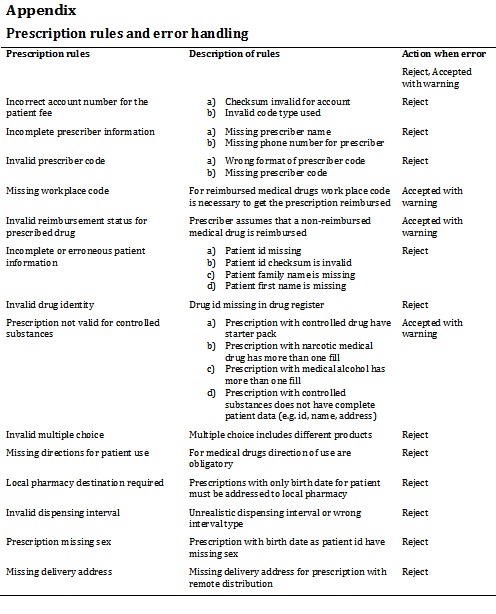

Supplement: Supplementary file 1 [file ijmr_v1i2e17_app1.jpg]
